# Hematopoietic Cytokine Gene Duplication in Zebrafish Erythroid and Myeloid Lineages

**DOI:** 10.3389/fcell.2018.00174

**Published:** 2018-12-20

**Authors:** Jana Oltova, Ondrej Svoboda, Petr Bartunek

**Affiliations:** ^1^Department of Cell Differentiation, Institute of Molecular Genetics of the ASCR, v.v.i., Prague, Czechia; ^2^Department of Cellular and Molecular Medicine, University of California, San Diego, La Jolla, CA, United States

**Keywords:** zebrafish, hematopoiesis, cytokine, genome duplication, myelopoiesis, erythropoiesis

## Abstract

Hematopoiesis is a precisely orchestrated process regulated by the activity of hematopoietic cytokines and their respective receptors. Due to an extra round of whole genome duplication during vertebrate evolution in teleost fish, zebrafish have two paralogs of many important genes, including genes involved in hematopoiesis. Importantly, these duplication events brought increased level of complexity in such cases, where both ligands and receptors have been duplicated in parallel. Therefore, precise understanding of binding specificities between duplicated ligand-receptor signalosomes as well as understanding of their differential expression provide an important basis for future studies to better understand the role of duplication of these genes. However, although many recent studies in the field have partly addressed functional redundancy or sub-specialization of some of those duplicated paralogs, this information remains to be scattered over many publications and unpublished data. Therefore, the focus of this review is to provide an overview of recent findings in the zebrafish hematopoietic field regarding activity, role and specificity of some of the hematopoietic cytokines with emphasis on crucial regulators of the erythro-myeloid lineages.

## Introduction

Hematopoiesis, the multistep process of formation and turnover of blood cells, is precisely regulated by an array of extrinsic and intrinsic factors (Kaushansky, 2006). Extrinsic factors include cytokines and growth factors that bind to their corresponding receptors and in turn activate intracellular signaling molecules that further modulate cellular responses, mainly by controlling activity of different transcriptional activators or repressors. The process of hematopoiesis begins already in early development, where red blood cells and macrophages are formed in a primitive wave to provide necessary support for the developing embryo. Later in development, the whole system is largely driven by proliferation, self-renewal, and differentiation of lineage restricted progenitors as well as hematopoietic stem cells (HSCs) and hematopoietic multipotent progenitor cells (MPPs) with reduced self-renewal capabilities. All erythroid and myeloid cells derive from HSCs with the exception of some tissue resident macrophages which are independent on HSC input (Ginhoux et al., 2010).

According to the classical hierarchical model of definitive vertebrate hematopoiesis (Akashi et al., 2000), the HSCs asymmetrically lose their long term self-renewing capabilities to form MPPs that further give rise to common lymphoid and common myeloid progenitors. The common myeloid progenitors afterward differentiate into bipotent megakaryocyte-erythrocyte progenitors (MEPs) and restricted common myelo-monocytic progenitors (CMPs). In this review, we will focus on cytokines that regulate formation and maintenance of these erythro-myeloid hematopoietic lineages.

**FIGURE 1:**
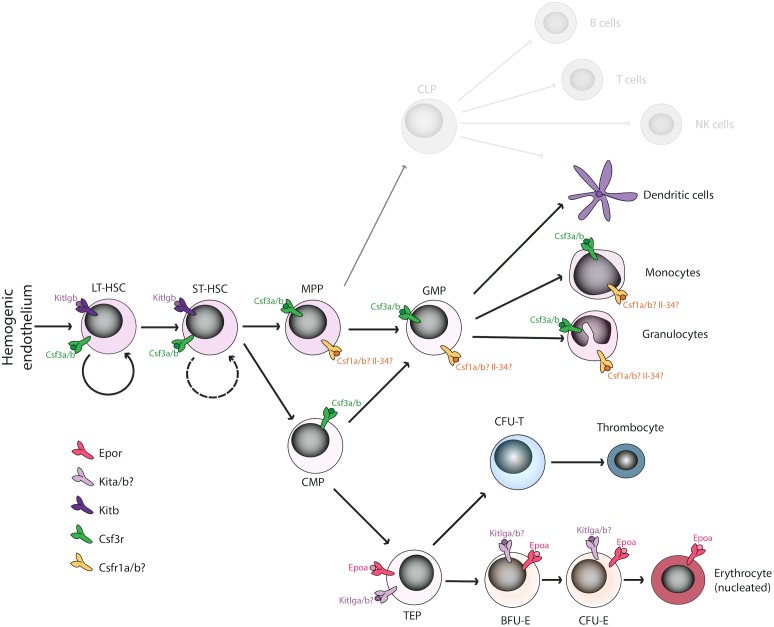
Overview of duplicated hematopoietic cytokines and receptors regulating the erythro-myeloid lineage in *Danio rerio*. Various cytokine receptors have been proposed to act at the level of hematopoietic stem cells (Kitb, Csf3r), the myeloid lineage (Csf1ra/b, Csf3r), or the erythroid lineage (Epor, Kita/b) in *D. rerio* binding the corresponding ligands. The function of missing cytokines (Il-3, Il-5, GM-CSF, Fli3) is most likely compensated by other factors. Some duplicated paralogs have undergone sub-specification (e.g., Kitlga/b), whereas some other seem to have lost their function in hematopoiesis (e.g., Epob). LT-HSC, long-term hematopoietic stem cells; ST-HSC, short-term hematopoietic stem cells; MPP, multipotent progenitor cells; GMP, granulocyte-macrophage progenitors; CMP, common myeloid progenitors; TEP, thrombo-erythroid progenitors; BFU-E, burst forming units-erythroid; CFU-E, colony forming units-erythroid.

Hematopoiesis is well conserved throughout the vertebrates, with all the major blood lineages – myeloid, erythroid and lymphoid – conserved from fish to men. Importantly, also the sequential waves of developing blood cells during ontogenesis are present during the development of the zebrafish embryo, leading finally to a fully fledged adult hematopoietic system, as has been described in other vertebrates. However, some differences between mammalian and non-mammalian hematopoiesis do exist (Svoboda and Bartunek, 2015), particularly in erythro-megakaryocytic lineages. In mammals, bi-potent cells termed megakaryocyte-erythrocyte progenitors (MEPs) give rise to either endoreduplicated megakaryocytes (Svoboda et al., 2014) that serve as a precursor for platelet biogenesis, or to enucleated erythrocytes (Muir and Kerr, 1958; Simpson, 1967). On the other hand, non-mammalian vertebrates possess bi-potent thrombocyte-erythrocyte progenitors (TEPs) instead that differentiate into functional homologs of platelets, termed thrombocytes or to nucleated erythrocytes (Figure 1; Ratnoff, 1987; Schneider and Gattermann, 1994; Svoboda et al., 2014). Besides these differences, the rest of the hematopoietic differentiation tree is well conserved in all vertebrate animals.

Hematopoietic cytokines signal via their cognate receptors to drive target cell proliferation and/or differentiation. In general, cytokines are pleiotropic in their function and for this reason also many factors regulating erythro-thrombocytic differentiation have broader effect on all hematopoietic lineages (Nicola, 1994).

In vertebrates, the major cytokines regulating red blood cell development from bipotent TEPs or MEPs through committed burst forming units-erythroid (BFU-E), colony forming units-erythroid (CFU-E) and erythroblasts, are erythropoietin (EPO) and stem cell factor (SCF, or KIT ligand, KITLG). On the other hand, thrombopoietin (TPO) is the key mediator of thrombocyte or platelet formation from TEPs/MEPs and is also responsible for platelet formation from polyploid megakaryocytes (Kaushansky et al., 1995; Kato et al., 1998). Other important erythro/thrombocytic regulators that promote self-renewal of erythroid progenitors or their differentiation include insulin (INS) and insulin-like growth factor (IGF1) (Miyagawa et al., 2000). Moreover, transforming growth factor α (TGFα) and TGFβ family members (Krystal, 1994; Huber et al., 1998; Gandrillon et al., 1999; Fuchs et al., 2002; Harandi et al., 2010), interleukin 3 (IL3), and fibroblast growth factor 2 (FGF2) (Bartunek et al., 2002) also play a crucial role in this process.

In thrombocytic differentiation, TPO interacts with and activates its cognate receptor, TPOR (c-MPL) (de Sauvage et al., 1994; Alexander, 1999b) and this signaling has been shown to be necessary for proper thrombopoiesis (Alexander, 1999a). This signaling is complemented by IL12 and SDF1, necessary for proper megakaryocytic maturation and proper platelet formation (Gordon and Hoffman, 1992). Function of these lineage-restricted factors is complemented by other regulators, especially interleukins (IL3, IL6, IL11), G-CSF, GM-CSF (McNiece et al., 1991; Gordon and Hoffman, 1992) and SCF (Steinlein et al., 1995; Broudy, 1997) that can enhance both erythroid and thrombocytic differentiation.

The CMPs also give rise to other substantial myelo-monocytic cell types – granulocytes, monocytes/macrophages and dendritic cells (Akashi et al., 2000), whose proliferation and differentiation from hematopoietic stem and progenitor cells (HSPCs) is regulated by macrophage colony-stimulating factor (M-CSF, or CSF1), granulocyte-macrophage CSF (GM-CSF, or CSF2), granulocyte CSF (G-CSF, or CSF3) and interleukin 3 (IL3) (Metcalf and Nicola, 1983; Metcalf, 1985; Migliaccio et al., 1991; Lieschke et al., 1994; Liu et al., 1996). These cytokines act via their cognate receptors – M-CSF receptor (M-CSFR, or CSF1R), G-CSF receptor (G-CSFR, or CSF3R), GM-CSF receptor (GM-CSFR, or CSF2RA) and interleukin 3 receptor (IL3RA), respectively.

Due to poor sequence homology between teleost and mammalian cytokines, the mammalian cytokines generally do not cross-react with zebrafish hematopoietic cells (Stachura et al., 2009, 2011, 2013; Svoboda et al., 2014) and for the same reason, the identification of zebrafish cytokine orthologs has been challenging. However, many successful attempts of identifying, generating and using recombinant zebrafish cytokines have been reported in recent years (Stachura et al., 2009, 2011, 2013; Svoboda et al., 2014).

Due to an extra round of whole genome duplication (WGD) during the evolution of teleost fish, which occurred 320–350 million years ago (Hoegg et al., 2004; Amores et al., 2011), zebrafish possess multiple paralogs of many important genes. After WGD, duplicated paralogs are often lost through a process of pseudogenization, where detrimental mutations accumulate in the duplicated gene (Nei and Roychoudhury, 1973; Takahata and Maruyama, 1979; Watterson, 1983). Alternatively, the two paralogs of the ancestral gene can be retained and either acquire new functions (i.e., neofunctionalization) or split the original function between the two paralogs (i.e., subfunctionalization) (Force et al., 1999).

Importantly, the event of duplication brought an increased level of complexity in such cases when both ligands and receptors have been duplicated (and retained) in parallel. Therefore, precise understanding of binding specificities between duplicated ligand-receptor signalosomes, as well as an understanding of their differential expression, will provide an important basis for future studies to better understand evolution of the vertebrate genome. Although many recent studies in the field have partly addressed functional redundancy or sub-specialization of some of these duplicated paralogs (Hultman et al., 2007; Wang et al., 2008; Stachura et al., 2013; Butko et al., 2015), there are still many unknowns.

Understanding the precise relationship between mammalian and non-mammalian hematopoiesis may have an important impact on general hematopoiesis research. Due to the interference with sophisticated mammalian megakaryocytic and erythroid enhancements, the employment of mammalian model organisms brings only partial success in the quest of identifying novel key regulators of cell fate determination. Once we have detailed understanding of cytokines and the receptors driving hematopoiesis in zebrafish, we can overcome these obstacles and efficiently utilize this non-mammalian model organism, instead.

## Duplicated Erythro-Myeloid Cytokine Genes in Zebrafish

### Epoa/b

Erythropoietin (EPO) is the major regulator of erythropoiesis, mediating self-renewal, survival and differentiation via the Epo receptor (EPOR) (Krantz, 1991). EPOR homodimerization mediated by EPO binding leads to auto-phosphorylation of JAK2 that is bound to Box1/2 and that in turn phosphorylates the receptor itself as well as other signaling molecules such as STAT5 (signal transducers and activators of transcription) transcription factor, PI-3K (phosphatidylinositol 3-kinase) and mitogen-activated protein kinase (MAPK) (Drachman and Kaushansky, 1997; Constantinescu et al., 1999).

We previously identified two copies of the epo gene in zebrafish, termed epoa and epob. Although zebrafish epoa probably plays a similar role as its mammalian ortholog, the epob has not been studied so far and it is likely that it formed a pseudo-gene without having any biological role. Zebrafish erythropoietin encoded by the epoa gene is a crucial cytokine for erythroid cell development and maintenance (Krystal, 1994) and it has been shown to stimulate proliferation, and differentiation of erythroid cells (Paffett-Lugassy et al., 2007; Stachura et al., 2011). Moreover, recombinant zebrafish Epoa has been shown to expand and differentiate erythroid cells *ex vivo* (Stachura et al., 2009) and in combination with other factors such as Gcsfa or Tpo, and it promotes multilineage erythro-myeloid hematopoiesis in semisolid media (Stachura et al., 2011; Svoboda et al., 2014). To date, a single erythropoietin receptor has been identified in zebrafish (Paffett-Lugassy et al., 2007).

### Kitlga/b

KIT ligand (KITLG) is a classic example of a cytokine acting on many different levels. KIT signaling plays an important role in a variety of tissues and cells, such as HSCs and germ stem cells. Moreover, KIT also plays a significant role during erythro-myelopoiesis (Hayman et al., 1993), neurogenesis and pigmentation, and mutations in this gene have been reported in many types of cancers including erythroleukemia (Kosmider et al., 2005; Abbaspour Babaei et al., 2016). Binding of KITLG to its cognate receptor (KIT) – member of the receptor tyrosine kinase type III family (RTKIII) – promotes various signaling cascades. Ligand-based activation of Kit triggers auto-phosphorylation and activation of PI-3K, MAPK, SRC, but also JAK kinase pathways (Ashman, 1999; Abbaspour Babaei et al., 2016). Notably, KITLG exists in two forms *in vivo* – transmembrane, which seems to be important to regulate stem cells in their niches, and soluble, that affects distant tissues (Ashman, 1999).

Interestingly, both Kit and Kitlg have been duplicated in teleost to form two receptors (Kita and Kitb) and two ligands (Kitlga and Kitlgb). This duplication of the whole ligand-receptor signalosome is relatively uncommon and raises many questions about diversification of both ligands and receptors as well as their binding specificities for each other. These questions have been poorly studied so far and very little is known about Kit involvement during zebrafish hematopoiesis. So far, it has been shown that both kitlg paralogs might have subspecialized during teleost phylogenesis. Kita is expressed in neural crest, lateral line or in the notochord. Similar to their mammalian counterparts, both Kitlga as well as Kita are involved in melanogenesis, since overexpression of kitlga results in a hyper pigmentation phenotype (Hultman et al., 2007), whereas kita receptor mutants (sparse) show severe pigmentation defects (Parichy et al., 1999). On the contrary, the second zebrafish Kit paralog, Kitb, does not seem to play a role during melanogenesis. It has been shown to be expressed in neural tube and otic vesicles (Mellgren and Johnson, 2005).

As mentioned above, Kit signaling has been poorly studied in the hematopoietic context and there are only two studies that present any possible Kit involvement in these processes. It has been shown that even though the kita is expressed in hematopoietic tissues, surprisingly and in contradiction to the other vertebrate models, hematopoiesis does not seem to be affected in the kita receptor (sparse) mutants (Parichy et al., 1999). So far, the only studies demonstrating any potential importance of Kit signaling during hematopoiesis in zebrafish at present are a mild increase of HSCs upon overexpression of kitlgb (Mahony et al., 2016) and decrease of HSCs upon downregulation of kitb (Mahony et al., 2018).

### Csf3a/b

Colony stimulating factor 3, CSF3, also known as granulocyte colony stimulating factor (GCSF), is a cytokine crucial for proliferation, differentiation and survival of monocytes, macrophages and neutrophilic granulocytes (Metcalf and Nicola, 1983; Nicola et al., 1983, 1985; Lieschke et al., 1994; Liu et al., 1996). CSF3 binds to its cognate receptor, CSF3R, activating signaling cascades including JAK2/STAT5 and MAPK pathway, important for neutrophil production during both steady state and emergency hematopoiesis (Touw and van de Geijn, 2007).

Two paralogs of Csf3, the major regulator of granulocytic, monocytic and megakaryocytic differentiation, have been reported in zebrafish with a slightly diverged function. Based on extensive synteny analysis, it has been suggested that chromosomal regions harboring Csf3a/b share common ancestral origin and probably emerged from a chromosome/genome duplication event (Stachura et al., 2013). Although like in mammals, both zebrafish Csf3 paralogs stimulate granulocytic differentiation (Nicola et al., 1983, 1985) and monocyte/macrophage differentiation (Migliaccio et al., 1988), they appear to play a broader role in hematopoiesis, including HSCs specification and expansion. Both paralogs differ in the levels of spatio-temporal expression during development and in the adult animals. Csf3a expression is low in early development, rising gradually over time, whereas Csf3b is highly expressed starting from 6 hpf but its levels decrease over time. Despite these differences, overexpression of both ligands during development indicates redundant functions. Slight differences in binding kinetics to Csf3r, indicate another possible mechanism that controls spatio-temporal activity of both zebrafish Csf3 paralogs (Stachura et al., 2013).

High csf3b expression has been detected in the kidney, the main site of hematopoiesis in zebrafish, as well as testes, skin and gills. Csf3a has been detected at lower levels in these tissues, with high expression in heart and spleen. Although the differences in tissue expression might indicate that the major player could be csf3b, both of these ligands retain the ability to differentiate myeloid progenitors. As in mammals, Csf3 is important for both primitive and definitive waves of generation of myelomonocytic cells (Stachura et al., 2013).

These findings suggest that during vertebrate evolution, Csf3 was involved in many levels of hematopoiesis, but after the radiation of mammals other specialized cytokines evolved and have likely taken over the function of Csf3 (Avery et al., 2004; Huising et al., 2006). Csf3 probably represents an ancient cytokine whose functions were diversified in evolution following duplication events (Stachura et al., 2013).

### Csf1a/b and IL34

In mammals, CSF1 is the major regulator of many myeloid cells, such as monocytes, macrophages, dendritic cells, microglia, osteoclasts, or Langerhans and Kupffer cells, and it also plays an important role in disease development (Hamilton, 2008; Hamilton and Achuthan, 2013). The ligand binds to its specific receptor (CSF1R), which is another member of RTKIII family, and activated CSF1R further promotes JAK2/STAT5, PI3K, and MEK signaling (Pixley and Stanley, 2004; Martinez and Gordon, 2014). It has been shown that CSF1 is not the only ligand to bind and activate this receptor. Alternatively, it can be activated also by IL34 in certain tissues – Langerhans cells and microglia inside mouse brain (Greter et al., 2012; Wang et al., 2012).

Similarly to other cytokines described, the CSF1 has been identified in the form of two paralogs in zebrafish (csf1a/b) (Wang et al., 2008). Along with two csf1 receptors (csf1ra/b) found in fish, this provides another relatively unique example of duplication of the whole receptor-ligand signalosome (Braasch et al., 2006). Regarding the IL34 that has been similarly reported to exist in two copies in salmon (Pagan et al., 2015), only a single il34 gene has been identified in zebrafish so far.

Zebrafish Csf1a and Il34 signaling has been shown to play a role in microglia development in the retina (Huang et al., 2012) as well as in pigment pattern formation during development (Patterson et al., 2014) (Csf1a only). Supporting this, the csf1ra mutant fish (panther) have decreased numbers of microglia and macrophages (Herbomel et al., 1999; Pagan et al., 2015). Il34 has recently been shown to regulate distribution of yolk sac macrophages and microglial precursors and seeding of the brain in zebrafish embryos (van Ham et al., 2018; Wu et al., 2018). On the other hand, any information about the functional role of csf1rb are missing.

### Examples of Potentially Missing Cytokine Genes

Genome evolution in the teleost fish did not only bring another rounds of duplication, but it also brought many losses of individual genes or clusters of genes including some of the class I cytokine family members (Liongue and Ward, 2007). This includes a missing cluster of il3 family genes with the disappearance of ligands and receptors for il3, il5, and gmcsf. In mammals and birds, these factors are responsible for the maintenance of myelo-monocytic lineages and their loss in teleost indicates that they were possibly substituted by other newly duplicated genes (Stachura et al., 2013).

Another example of a cytokine potentially missing in the zebrafish genome is flt3l, an important regulator of HSCs and myeloid cells (Guermonprez et al., 2013; Jacobsen et al., 2016; Tornack et al., 2017). However, the possibility exists that it has not yet been identified due to sequence divergence. This hypothesis is supported by the fact that flt3l is present both in mammals and even some lower vertebrates including *Latimeria* and elephant shark (Tan et al., 2012) and its cognate receptor, Flt3, is expressed in the developing zebrafish embryo and adult HSCs and monocytes (He et al., 2014; Macaulay et al., 2016; Tang et al., 2017).

## Conclusion

The whole-genome duplication in teleost fish raises interesting questions regarding hematopoietic cytokine sub-specialization, redundancy and gain/loss of function. In this review, we have reviewed particular examples of all of these events, discussing epob loss of function, sub-specialization of kitlga/b, or the redundancy between csf3a/b (Figure 1). We hypothesize that some specific gene duplications might even have enabled or compensated for the loss of some specific genes in the zebrafish genome (GM-CSF/IL3 cluster).

Although several reports have addressed the question of duplicated cytokines in zebrafish, many functional links are still missing, especially the ligand receptor specificities in cases, when the whole ligand-receptor signalosome has been duplicated (e.g., Kitlg/Kit, Csf1/Csf1r). Moreover, functions of many hematopoietic cytokines have yet not been elucidated. One example is IL11, a crucial regulator of megakaryocyte maturation (Paul et al., 1990) that binds to IL11Rα and gp130, activating the JAK/STAT pathway (Heinrich et al., 2003). Two paralogs of il11 have been identified in teleost (Huising et al., 2005); however, data indicating respective functions of each of the paralogs are still missing.

Zebrafish is a powerful model organism for studies of hematopoietic cell maintenance and differentiation both in the course of development and in the adult animal using the wide range of available *in vivo* and *ex vivo* tools. Therefore, it is very important to understand the precise function of each of the paralogs and elucidate the functions of the yet uncharacterized cytokines that would enable more complex experiments elucidating the fine details of hematopoietic regulatory mechanisms.

## Author Contributions

All authors listed have made a substantial, direct and intellectual contribution to the work, and approved it for publication.

## Conflict of Interest Statement

The authors declare that the research was conducted in the absence of any commercial or financial relationships that could be construed as a potential conflict of interest.
